# The Protective Effect of the Crosstalk between Zinc Hair Concentration and Lymphocyte Count—Preliminary Report

**DOI:** 10.3390/life14050571

**Published:** 2024-04-29

**Authors:** Tomasz Urbanowicz, Anetta Hanć, Jolanta Tomczak, Michał Michalak, Anna Olasińska-Wiśniewska, Patrycja Rzesoś, Mateusz Szot, Krzysztof J. Filipiak, Beata Krasińska, Zbigniew Krasiński, Andrzej Tykarski, Marek Jemielity

**Affiliations:** 1 Cardiac Surgery and Transplantology Department, Poznan University of Medical Sciences, Dluga 1/2, 61-848 Poznan, Poland; turbanowicz@ump.edu.pl (T.U.); annaolasinska@ump.edu.pl (A.O.-W.);; 2 Department of Trace Analysis, Faculty of Chemistry, Adam Mickiewicz University, 61-614 Poznan, Poland; 3 Department of Vascular, Endovascular Surgery, Angiology and Phlebology, Poznan University of Medical Science, Dluga 1/2, 61-848 Poznan, Poland; jolanta.tomczak@usk.poznan.pl (J.T.);; 4 Department of Computer Science and Statistics, Poznan University of Medical Sciences, Rokietnicka 7, 60-806 Poznan, Poland; michal@ump.edu.pl; 5 Faculty of Medicine, Poznan University of Medical Sciences, 61-701 Poznan, Poland; patrycja.rzesos@usk.poznan.pl (P.R.); mateo.szot@gmail.com (M.S.); 6 Institute of Clinical Science, Maria Sklodowska-Curie Medical Academy, 00-136 Warsaw, Poland; krzysztof.filipiak@uczelniamedyczna.com.pl; 7 Department of Hypertensiology, Angiology and Internal Medicine, University of Medical Sciences, Dluga 1/2, 61-848 Poznan, Poland; beata.bkrasinska@gmail.com (B.K.);

**Keywords:** Zn 1, trace elements 2, lymphocyte 3, CAD 4, carotid 5, Cr 6, Cu 7

## Abstract

Background: An imbalance between pro- and anti-inflammatory mechanisms is indicated in the pathophysiology of atherosclerotic plaque. The coronary artery and carotid disease, despite sharing similar risk factors, are developed separately. The aim of this study was to analyze possible mechanisms between trace element hair–scalp concentrations and whole blood counts that favor atherosclerotic plaque progression in certain locations. Methods: There were 65 (36 (55%) males and 29 (45%) females) patients with a median age of 68 (61–73) years enrolled in a prospective, preliminary, multicenter analysis. The study group was composed of 13 patients with stable coronary artery disease (CAD group) referred for surgical revascularization due to multivessel coronary disease, 34 patients with carotid artery disease (carotid group) admitted for vascular procedure, and 18 patients in a control group (control group). Results: There was a significant difference between the CAD and carotid groups regarding lymphocyte (*p* = 0.004) counts. The biochemical comparison between the coronary and carotid groups revealed significant differences regarding chromium (Cr) (*p* = 0.002), copper (Cu) (*p* < 0.001), and zinc (Zn) (*p* < 0.001) concentrations. Spearman Rank Order Correlations between lymphocyte counts and trace elements in the analyzed groups were performed, revealing a strong correlation with zinc (R = 0.733, *p* < 0.001) in the control group (non-CAD, non-carotid). Conclusion: Significant differences in hair–scalp concentrations related to atherosclerosis location were observed in our analysis. The interplay between zinc concentration and lymphocyte count may play a pivotal role in cardiovascular disease development.

## 1. Introduction

Among various trace elements’ significance, the modulatory role of zinc has been reported in immunological mechanisms [1]. It affects multiple aspects of immune system functions, from cell development to the mediation of their activation [2]. Zinc-dependent proper cytokine function and secretion indicate its role in innate and adaptive immunological responses [3]. Zinc is believed to be a balancing agent enabling a sufficiently strong but not overshooting immunological response [1]. Zalewski et al. [4] discussed the significance of Zn on endothelial function by nitric oxide (NO) generation. NO is crucial for large and microvascular vessel integrity and function. In in vitro models, the modulatory role of zinc for T regulatory cells and a significant reduction in interleukin expression were observed [5].

There is accumulating evidence that immunological activation is imperative for atherosclerosis development and progression [6] and that cardiovascular adverse events increase this threat [7]. The imbalance between pro- and anti-inflammatory mechanisms is indicated in the pathophysiology of atherosclerotic plaque [8]. Recent reports highlighted this phenomenon as a potential target for new therapies identification [9]. The inflammatory activation was found to be related not only to chronic progression [10] but also to acute cardiovascular events [11,12] and was presented as an independent potential survival modulator [13]. Zernecke et al. [14] in their analysis presented a strong atheroprotective role of innate lymphoid cells-2. Immune system activation possesses contradictory properties as it may play a fundamental role in different stages of plaque progression or activate protective mechanisms against atherosclerosis development. Thus, it is essential to have an in-depth understanding of the effectors of its initiation and progression.

Coronary artery and carotid artery diseases, despite sharing similar risk factors, develop independently [15,16,17]. Atherosclerotic plaques tend to occur in specific locations; however, the relationships or interactions between various vascular systems remain unknown. It seems reasonable that some mechanisms exist that favor atherosclerotic plaque progression in certain locations. Importantly, the development of atherosclerosis is multifactorial, and inflammatory activation may be triggered by different agents. 

The aim of our analysis was to find a possible relation between zinc concentrations and inflammatory indices that may vary between healthy controls and patients presenting with coronary or carotid diseases.

## 2. Materials and Methods

### 2.1. Patients

There were 65 (36 (55%) males and 29 (45%) females) consecutive patients enrolled in a prospective analysis. The study group was composed of 13 patients with stable multivessel coronary artery disease (CAD group) referred for surgical revascularization, 34 patients with carotid artery disease (carotid group) admitted for vascular procedure, and 18 patients in a control group (control group) presenting with normal coronary angiograms and carotid doppler ultrasounds. 

The coronary artery disease (CAD) group was composed of 11 men and 2 women with a median age of 70 (59–73) years admitted to the Cardiac Surgery Department for surgical revascularization. There were 6 patients diagnosed with left main coronary artery disease, 5 patients with three-vessel disease, and two more patients referred for surgery due to two-vessel disease. They were characterized by the following co-morbidities: hypercholesterolemia (*n* = 13, 100%), arterial hypertension (*n* = 13, 100%), diabetes mellitus (*n* = 10, 77%), and atrial fibrillation (*n* = 5, 38%). On preoperative echocardiographical examination, the left ventricular ejection fraction was estimated to be 55 (50–60%) with a left ventricular diastolic diameter of 55 (53–59) mm.

The carotid group was composed of 34 participants (including 15 males and 19 females) with a median age of 68 (64–73) years referred for carotid surgery at the Vascular Surgery Department. The culprit lesions were located in the right (*n* = 20) or left (*n* = 14) carotid artery. Co-existing co-morbidities, including arterial hypertension (*n* = 29, 85%), hypercholesterolemia (*n* = 29, 85%), diabetes mellitus (*n* = 9, 26%), and chronic obstructive pulmonary disease (COPD) (*n* = 1, 3%) were reported by the enrolled patients.

The control group was composed of 18 (10 men and 8 women) patients with a median age of 66 (61–71) years who were diagnosed with hypertension at the Internal Disease Department, with confirmed normal coronary angiography and ultrasound carotid doppler. Arterial hypertension (*n* = 15, 83%), hypercholesterolemia (*n* = 12, 67%), and diabetes mellitus (*n* = 2, 11%) were diagnosed as co-morbidities. The detailed characteristics are presented in Table 1. 

### 2.2. Hair–Scalp Trace Element and Blood Sample Measurements

On admission, hair and blood samples were collected. Patients’ data concerning demographic and clinical data were gathered. The obtained biochemical results were scrutinized together with patients’ characteristics. 

All samples of peripheral blood were collected under equal conditions. The inflammatory system activation markers were obtained from blood analysis including neutrophil count, lymphocyte count, monocyte count, haemoglobin, platelet count, and relevant indexes—the aggregate index of systemic inflammation (AISI), neutrophil-to-lymphocyte ratio (NLR), monocyte-to-lymphocyte ratio (MLR), platelet-to-lymphocyte ratio (PLR), systemic inflammatory index (SII), serum creatinine, and lipid profiles—were immediately measured with a routine haematology analyzer (Sysmex Euro GmbH, Norderstedt, Germany). Glomerular filtration rate (GFR) was estimated by the simplified formula of the Modification of Diet in Renal Disease (MDRD).

Hair samples (0.5 g by the patient) were cut in a similar manner from the scalp, just above the patient’s neckline, with the use of titanium scissors. All the samples were stored in plastic containers. They were kept at room temperature until the analysis was performed. 

Hair samples were routinely washed thoroughly, stirring with acetone, deionized water, 0.5% Triton X-100 solution, and deionized water. All the hair samples were, thereafter, dried and cut into smaller pieces. The next step involved sample digestion in a high-pressure closed microwave system (Ethos One, Milestone, Sorisole, Italy). Briefly, 200 mg of already prepared samples was accurately weighed into the microwave vessels and then 3 mL of 65% HNO_3_ and 1 mL of 30% H_2_O_2_ were added. After that, samples were diluted to exactly 50 mL and were then ready for the measurement process. An inductively coupled plasma mass spectrometer (ICP-MS 7100x Agilent, Santa Clara, CA, USA) was used for the detection of the following elements—chrome (Cr), Mangan (Mn), copper (Cu), zinc (Zn), iron (Fe), lead (Pb), cadmium (Cd).

The instrumental parameters were optimized using a tuning solution (Agilent). Spectral interferences were reduced by a helium mode. The non-spectral and matrix interferences were reduced using an internal standards solution containing 10 µg/L Y and Tb introduced in parallel with all analyzed solutions.

### 2.3. Analytical Figures of Merit

The validity of the analytical method was assessed by analyzing the certified reference material (CRM) NCS ZC 81002b Human Hair (Beijing, China). The CRMs were digested according to the same procedure as that for the hair samples. Validation parameters such as linearity, precision, limit of detection (LOD), and trueness were evaluated. The linearity of the calibration curve was calculated as the correlation coefficient (R), the value of which was greater than 0.9996 for all analytes. The linear range of the calibration curve of elements was reached from the detection limit up to 100 μg/l. The LOD was defined as 3.3 s/b, where s is the standard deviation corresponding to 10 blank injections and b is the slope of the calibration graph. The LOD values were in the range of 0.006 µg/g for Cd to 10 µg/g for Ca. Precision values were calculated as the coefficient of variation (CV) (%), which ranged from 1.5% to 3.4% for all elements. Trueness was evaluated by applying the certified reference material and expressed as recovery values (%) ranging from 94% to 107%, respectively.

### 2.4. Statistical Analysis

The Shapiro–Wilk test was applied for the normality of the distribution of variables. The t-test, Cochran–Cox test, Mann–Whitney test, or Fisher’s exact test was used where applicable to compare the variables between the two groups. Spearman correlation analysis was used to describe the correlation between the variables. Uni- and multivariable models were used to predict either coronary or carotid disease. Receiver operator characteristic (ROC) analysis for zinc hair–scalp concentration for atherosclerosis prediction was carried out. Statistical analysis was performed using Statistica 13 by TIBCO. *p* values < 0.05 were considered statistically significant. 

## 3. Results

### 3.1. Coronary Artery Group

There were no perioperative deaths in this group and the median [IQR] hospitalization time after surgery was 8 days (6–10). All procedures were performed as off-pump surgery (beating heart surgery). The mean (SD) number of performed grafts was 2.3 (2–2.5). Ten patients underwent total arterial revascularization, including eight with two internal mammary arteries and one with three arterial grafts application (two mammary arteries and a left radial artery).

### 3.2. Carotid Group

There were no intra- nor postoperative deaths in this group and the median [IQR] hospitalization time after surgery was 2 days (2–3). Surgical procedures included carotid endarterectomy (*n* = 26, 76%) and open surgery corrections (*n* = 8, 24%). The postoperative time was uneventful. 

### 3.3. Laboratory Results

The laboratory analysis including peripheral blood counts performed to compare all groups. There was a significant difference between the CAD and carotid groups regarding lymphocyte counts (*p* = 0.004), large unstained cell (LUC) counts (*p* = 0.003), and the NLR (*p* = 0.049). Since the neutrophil count was insignificant, the NLR differences were related to lymphocytes.

Significant differences in mean corpuscular hemoglobin concentrations (MCHCs) were observed between peripheral blood analyses for the carotid and control groups (*p* < 0.001), as presented in Table 2.

In our analysis, we found significant differences in zinc hair–scalp concentrations between the healthy controls (105 (0–127) mg/L), CAD group (45.5 (0.0–155.4) mg/L) (*p* < 0.001), and carotid group (190 (155–225) mg/L), respectively. Interestingly, the atherosclerosis plaque location significantly differentiated both analyzed groups (45.4 (0.0–155.4 mg/L) vs. 190 (155–225) mg/L) (*p* < 0.001).

In the analysis, zinc hair–scalp concentration was detected as the only statistically significant trace element between the healthy and CAD groups (105.0 (0.0–127) mg/L vs. 45.4 (0.0–155.4) mg/L) *p* < 0.001).

We noticed significant differences in hair trace element concentrations between the CAD and carotid groups regarding chromium (1.65 (1.08–7.56) mg/L vs. 8.3 (5.3–12.7) mg/L) (*p* = 0.002), copper (17.4 (12.2–21.8) mg/L vs. 52.2 (38.0–80.0) mg/L) (*p* < 0.001), and zinc (105.0 (0.0–127) mg/L vs. 190 (155–225) mg/L) (*p* < 0.001).

The control and carotid group analysis revealed significant differences regarding chromium (1.86 (1.21–3.61) mg/L vs. 8.3 (5.3–12.7) mg/L) (*p* < 0.001), zinc (105 (0.0–127) mg/L vs. 190 (155–225) mg/L) (*p* < 0.001), and copper (23.5 (19.1–32.6) mg/L vs. 52.2 (38.0–80.0) mg/L) (*p* = 0.013) hair concentrations.

**Figure 1:**
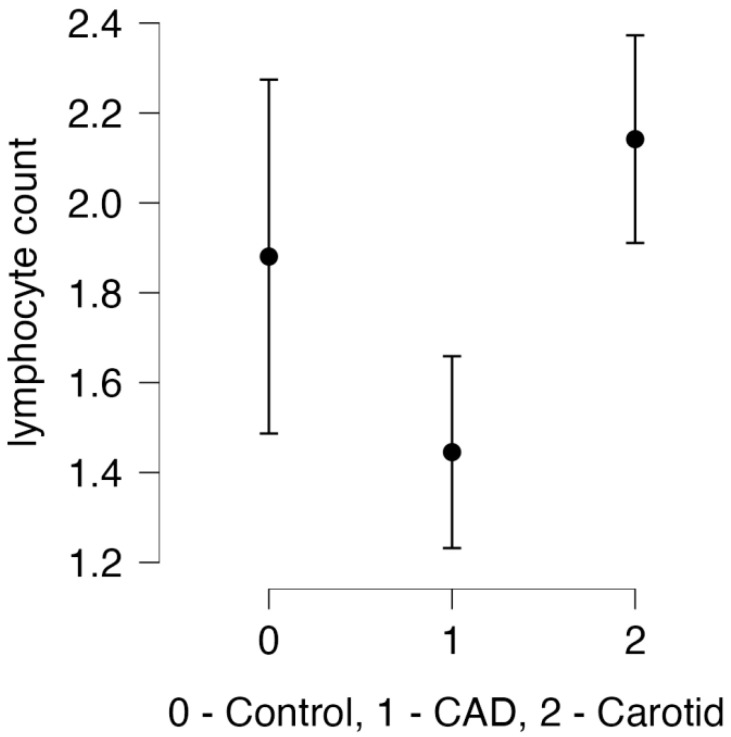
Lymphocyte counts in the presented groups, including significant differences between CAD and carotid groups (*p* = 0.004).

Thereafter, we focused on lymphocyte counts between the presented groups, as shown in Figure 1.

### 3.4. Trace Elements Results

The hair–scalp trace metal concentrations were analyzed in all groups. The biochemical comparison between the coronary and carotid groups revealed significant differences regarding chromium (*p* = 0.002), copper (*p* < 0.001), and zinc (*p* < 0.001) concentrations. In the control and CAD groups, trace element hair analysis presented significant differences related to zinc (*p* < 0.001). In the carotid group, compared with the control group, significantly different concentrations of chromium (*p* < 0.001), copper (*p* = 0.013), and zinc (*p* < 0.001) were found in hair samples, as presented in Table 2.

### 3.5. Correlation between Trace Elements and Lymphocytes

**Figure 2:**
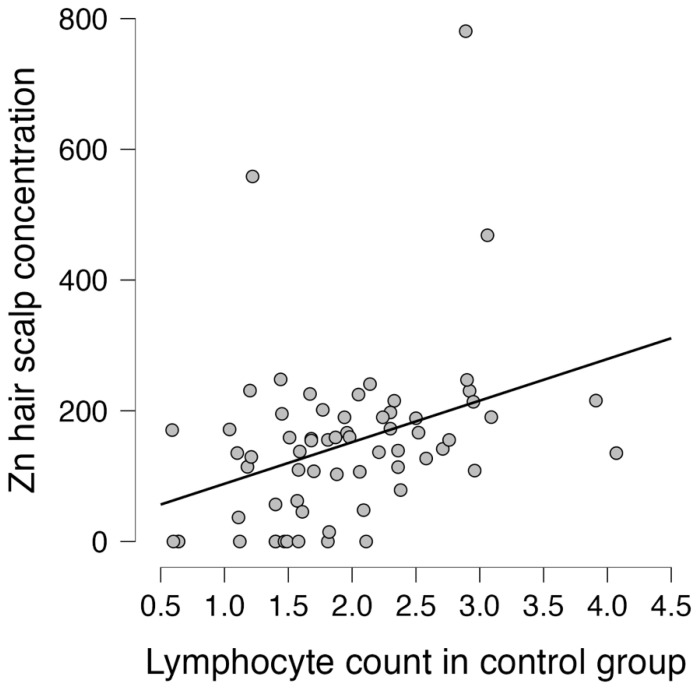
Correlation between zinc and lymphocyte count in the control group.

Spearman Rank Order Correlations between lymphocyte counts and trace elements in the analyzed groups were calculated as presented in Table 3, revealing a strong correlation with zinc (R = 0.733, *p* < 0.001) in the control group (non-CAD, non-carotid), as presented in Figure 2.

### 3.6. Multivariable Analysis

The uni- and multivariable analyses were performed for atherosclerosis prediction (either coronary or carotid disease). Demographic, clinical, and hair–scalp metal concentration data were taken into account in the analyses. Only trace elements representing significant differences between both groups were taken into account in the analyses (Zn, Cu), as presented in Table 4. Hair–scalp zinc concentration was found to be significant (OR 1.03, 95% CI: 1.01–1.05, *p* = 0.001) in the multivariable model for disease prediction.

### 3.7. Receiver Operator Curve Analysis

**Figure 3:**
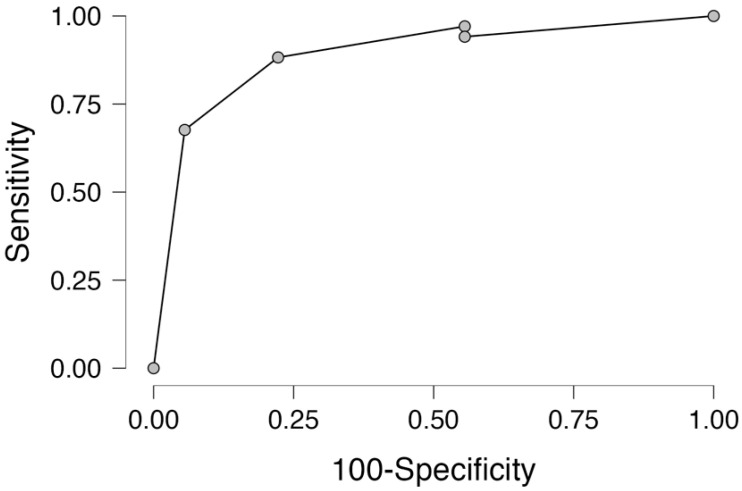
Receiver operator curve for prediction of atherosclerosis (CAD or carotid) in relation to hair–scalp zinc concentration.

Receiver operator curve (ROC) analysis for the prediction of atherosclerosis related to hair–scalp zinc concentration was performed. The whole study group (65 patients) was divided into two subgroups: control group vs CAD + carotid group. The ROC curve analysis related to zinc concentration presented an AUC of 0.913, yielding sensitivity of 88.2% and specificity of 58.8%, as presented in Figure 3.

## 4. Discussion

The results of our preliminary report indicate a strong correlation between hair–scalp zinc concentration and lymphocyte count in the control group. Zinc is a balancing agent that keeps the immune system in check. The main finding of this study is related to the possible guarding role of zinc in immunological responsiveness related to zinc–lymphocyte correlation.

Our analysis pointed out the role of zinc in the increased risk of either carotid or coronary disease occurrence in a multivariable model. The hair–scalp zinc concentrations were significant for atherosclerosis occurrence. Hair–scalp biochemical analysis was chosen as the source of trace element concentration analysis [17], though disparities between the organs were also reported [18].

The results of our analysis indicate a possible warden role of bodily zinc concentrations (measured in hair–scalp samples) and lymphocytes. As the control group (non-CAD and non-carotid) was characterized by moderate values of zinc, while the CAD and carotid groups presented low and high concentrations, respectively, we revealed a strong correlation with lymphocyte count. The presence of both mentioned factors may play a protective role against atherosclerosis development. This is the first study, to our best knowledge, that points out a possible supervisory mechanism between trace elements (zinc) and lymphocytes in atherosclerotic lesion formation. Our preliminary report suggests that any derangements in zinc body accumulation provoke the loss of a delicate balance between trace metal elements and inflammatory system components that may provoke atherosclerotic lesion progression. Our analysis did not focus on specific inflammatory reactions but only on lymphocyte count analysis and its relation to zinc concentration.

The correlation was lost when zinc disturbances were noticed in our analysis. Interestingly, low and high hair–scalp zinc concentrations were found in patients presenting with coronary and carotid artery disease, respectively. Despite non-significant differences in classical cardiovascular risk factors between the groups, culprit atherosclerotic lesions were found in different locations related to hair–scalp zinc concentrations. This phenomenon may be explained by two factors: zinc concentration and a lost correlation between this trace element and lymphocytes.

The results of our preliminary report may indicate that low zinc concentrations combined with a lost correlation in the lymphocyte–zinc axis may characterize increased risk of coronary artery development. The relationship between inflammatory activation and serum trace elements concentration in coronary artery disease was postulated in our previous analysis [19].

Our results are consistent with those of previous reports that suggested the beneficial effect of zinc supplementation on serum lipids and biomarkers of oxidative stress and inflammation in patients suffering from coronary artery disease [20]. In Banik et al.’s meta-analysis [21], the potential role of low levels of Zn in the pathogenesis of CAD was presented. The reduction in serum zinc ion concentrations and occurrence of coronary heart disease was investigated and presented by Meng et al. [22]. Liu et al. [23] proposed a zinc-*α*2-glycoprotein (ZAG) novel serum adipokine as a potential marker for coronary artery diagnosis markers. Decreased ZAG levels were independently associated with the presence of atherosclerotic epicardial lesions. Interestingly, in a recent study by Zang et al. [24], a possible relationship between low serum zinc concentrations and coronary artery disease risk combined with worse survival rates was suggested.

In our analysis, higher levels of hair–scalp zinc concentrations characterized patients with carotid disease. The results show that in coronary and carotid disease, zinc may play an important role in plaque location. In previous studies, higher zinc concentrations alongside fibrous aortic plaque were presented by Mendis [25]. Stadler et al. [26] in their analysis suggested that zinc in human atherosclerotic lesions binds to matrix components. Therefore, zinc may be associated with plaque stability by promoting accelerated calcification. The strong correlation between zinc fluorescence and atherosclerotic plaque content was presented by Kopriva et al. [27].

Multifaceted contributions of immune system compounds may drive atherosclerosis. The specific characteristics of immune cell dysregulation within atherosclerotic lesions are still poorly understood [28]. Lymphocytes’ role in atherosclerosis is claimed to be related to various aspects of plaque formation. Although it is largely unknown how they migrate to the lesion sites, some studies suggest a regulating role of chemokines and their receptors and L-selectins [29]. The suppressive role of zinc deficiency for altered lymphocyte maturation, activation, apoptosis, and lymphopenia suggesting an inefficient immune response was described [30,31]. Zinc disturbances also affect T lymphocyte maturation and function, including the regulation of many enzymes related to oxidative stress [32]. Jeong et al. [33] presented the association between the copper–zinc ratio in hair and the NLR, indicating an oxidative burden of individuals predisposed to obesity-related comorbidities. The results of in vitro studies suggest possible therapeutic options for zinc supplementation in various dysregulated immune conditions [34]. Zinc excess impairs immune cell-specific functions [35,36] and was in human studies related to decreased high-density lipoprotein cholesterol and impaired immune reactions [37]. 

The results of our preliminary report present a strong correlation between atherosclerosis (either coronary or carotid disease) and zinc concentration, as presented in the receiver operator curve analysis. Further studies are required on zinc hemostasis as it may play a pivotal role in lesion development. Based on these results, zinc could be taken into consideration for future cardiovascular risk modification protocols.

## 5. Study Limitation

This study was performed on a limited number of patients as a preliminary report. The analyzed lymphocyte counts did not indicate lymphocyte types nor their activation status, which may play crucial roles in the explanation of the pathophysiological mechanisms. The multivariable model was performed on data obtained from a relatively small group of patients, limiting the number of factors included in the analysis.

## 6. Conclusions

Significant differences in hair–scalp concentrations related to atherosclerosis location were presented in our analysis. The interplay between zinc concentration and lymphocytes may play a pivotal role in the absence of cardiovascular disease development. The zinc concentration disturbances followed by the loss of correlation between zinc and immune cells may help researchers understand the pathophysiology of atherosclerotic plaque location in human organisms.

## Figures and Tables

**Table 1:** Demographical and clinical characteristics.

	Group 1	Group 2	Group 3	*p*	*p*	*p*
	CAD = 13	Control = 18	Carotid = 34	1–2	1–3	2–3
Age (years, median, Q1–Q3)	70 (59–73)	66 (61–71)	68 (64–73)	1	1	0.938
BMI (median, Q1–Q3)	27 (25–29)	30 (27–34)	28 (26–30)	0.689	0.945	0.756
Sex (M (%), F (%))	11 (85)/2 (15)	10 (56)/8 (44)	15 (44)/19 (56)	0.044 *	0.051	0.324
Co-morbidities						
HA (*n*, %)	13 (100)	15 (83)	29 (85)	0.876	0.478	0.866
DM (*n*, %)	10 (77)	2 (11)	9 (26)	0.027 *	0.067	0.041 *
COPD (*n*, %)	2 (15)	0 (0)	1 (3)	0.233	0.532	0.272
Hypercholesterolemia (*n*, %)	13 (100)	12 (67)	29 (85)	0.784	0.407	0.148
Thyroid disease (*n*, %)	3 (23)	2 (11)	10 (29)	0.567	0.337	0.324
AF (*n*, %)	5 (38)	0 (0)	4 (12)	0.049 *	0.058	0.155
Smoking history (*n*, %)	8 (62)	8 (44)	18 (63)	0.453	0.867	0.703
Current smoker (*n*, %)	5 (38)	4 (22)	11 (32)	0.958	0.578	0.842

Abbreviations: AF—atrial fibrillation, BMI—body mass index, CAD—coronary artery disease, COPD—chronic obstructive pulmonary disease, DM—diabetes mellitus, F—female, HA—arterial hypertension, M—male. * statistically significant.

**Table 2:** Laboratory results and trace elements hair–scalp concentration [mg/L] comparison between groups.

Parameters	Group 1	Group 2	Group 3	*p*	*p*	*p*
(Median (Q1–Q3)	CAD *n* = 13	Control *n* = 18	Carotid *n* = 34	1–2	1–3	2–3
Laboratory:						
WBC (K/μL)	8.2 (7.1–8.8)	8.2 (6.3–9.9)	8.5 (6.8–9.8)	1	0.956	0.986
Neu (K/μL)	6.0 (4.9–6.6)	4.6 (3.8–7.3)	5.3 (4.4–6.9)	0.974	1	0.867
Lym (K/μL)	1.5 (1.2–1.6)	1.9 (1.4–2.4)	2.1 (1.6–2.8)	0.149	0.004 *	0.787
NLR	3.8 (3.5–4.5)	2.3 (1.8–3.7)	2.4 (1.9–3.6)	0.128	0.049 *	1
SII	888 (747–1288)	537 (406–921)	594 (452–920)	0.147	0.186	1
AISI	358 (314–513)	317 (147–844)	240 (169–468)	1	0.352	1
Mon (K/μL)	0.45 (0.4–0.5)	0.54 (0.40–0.68)	0.5 (0.4–0.6)	0.473	1	0.292
MLR	0.3 (0.2–0.4)	0.3 (0.2–0.4)	0.2 (0.2–0.3)	1	0.06	0.206
LUC (K/μL)	0.10 (0.09–0.12)	0.13 (0.10–0.15)	0.16 (0.12–0.19)	0.318	0.003 *	0.298
Rbc (M/μL)	4.86 (4.35–5.02)	4.76 (4.20–5.07)	4.54 (4.16–4.69)	1	0.122	0.537
Hb (mml/L)	9.0 (8.4–9.2)	8.8 (8.4–9.8)	8.7 (8.0–9.3)	1	0.989	1
Hct (%)	43 (40–45)	43 (41–49)	41 (38–44)	1	0.728	0.141
MCV (fL)	90 (88–92)	92 (88–97)	92 (89–96)	0.481	0.282	1
MCHC	20.7 (20.4–21.1)	20.4 (20.1–20.7)	21.0 (20.9–21.3)	0.475	0.088	<0.001 *
RDW (%)	13.6 (13.1–14.1)	13.7 (13.4–14.3)	13.7 (13.3–14.5)	1	1	1
Plt (K/uL)	214 (190–245)	218 (201–250)	248 (204–300)	1	0.495	0.545
MPV (fL)	8.2 (7.4–8.7)	8.7 (8.2–9.4)	8.2 (7.7–8.7)	0.181	1	0.17
Lipid profiles						
(median (Q1–Q3):						
Total (mmol/L)	3.99 (3.58–4.58)	4.1 (3.7–4.2)	3.69 (3.02–4.00)	1	0.866	0.187
HDL (mmol/L)	1.14 (0.91–1.35)	1.04 (0.94–1.46)	1.11 (0.92–1.41)	1	1	1
LDL (mmol/L)	2.43 (2.04–2.75)	2.34 (1.97–2.61)	2.25 (1.36–2.62)	0.967	0.897	0.901
GFR (median (Q1–Q3)						
(mL//min)	78 (73–86)	59 (53–76)	76 (70–85)	0.234	1	0.278
Hb1Ac (mean (SD) (%)	6.2 (0.2)	6.1 (0.3)	6.2 (0.3)	0.921	0.978	0.967
CRP						
(median (Q1–Q3)	3.5 (2.5–4.0)	2.0 (1.8–2.3)	1.9 (1.5–2.4)	0.125	0.098	0.93
Trace elements						
(median (Q1–Q3):						
Cr	1.65 (1.08–7.56)	1.86 (1.21–3.61)	8.3 (5.3–12.7)	1	0.002 *	<0.001 *
Mn	1.19 (0.79–2.09)	1.09 (0.28–2.09)	1.14 (0.69–1.83)	1	1	1
Fe	26.9 (15.8–34.9)	18.6 (9.9–40.7)	27.9 (17.9–37.4)	1	1	0.699
Cu	17.4 (12.2–21.8)	23.5 (19.1–32.6)	52.2 (38.0–80.0)	0.672	<0.001 *	0.013 *
Zn	45.4 (0.0–155.4)	105.0 (0.0–127)	190 (155–225)	<0.001 *	<0.001 *	<0.001 *

Abbreviations: AISI—aggregate inflammatory syndrome index; CAD—coronary artery disease; Cr—chromium; Cre—creatinine; CRP—C-reactive protein; Cu—copper; Fe—iron; GFR—glomerular filtration rate; Hb—hemoglobin; Hct—hematocrit; HDL—high-density lipoprotein; LDL—low-density lipoprotein; LUC—large unstained cell count; MCHC—mean corpuscular hemoglobin concentration; MCV—mean corpuscular volume; MLR—monocyte-to-lymphocyte ratio; Mn—manganium; Neutro—neutrophil count; NLR—neutrophil-to-lymphocyte ratio; Plt—platelet count; Rbc—red blood cell count; RDW—red blood cell distribution width; SII—systemic inflammatory index; WBC—white blood cell count; Zn—zinc. * statistically significant.

**Table 3:** Correlations between lymphocyte counts and hair–scalp trace elements.

	Group 1 CAD *n* = 13		Group 2 Control *n* = 18		Group 3 Carotid *n* = 34	
	Spearman R	*p*-Value	Spearman R	*p*-Value	Spearman R	*p*-Value
Lymphocyte count and Cr	0.063	0.837	−0.244	0.330	−0.040	0.822
Lymphocyte count and Mn	0.104	0.734	−0.442	0.066	0.143	0.421
Lymphocyte count and Fe	0.028	0.929	−0.233	0.351	0.040	0.822
Lymphocyte count and Cu	0.382	0.197	0.061	0.810	−0.052	0.770
Lymphocyte count and Zn	0.357	0.232	0.733 *	<0.001 *	0.120	0.499

Abbreviations: CAD—coronary artery disease, Cr—chromium, Cu—copper, Fe—iron, Mn—manganese, Zn—zinc. * statistically significant.

**Table 4:** Uni- and multivariable analyses for atherosclerosis prediction.

Parameters	Univariable Analysis			Multivariable Analysis		
	OR	95% CI	*p*	OR	95% CI	*p*
Demographic:						
1. Age	1.06	0.88–1.28	0.541			
2. Sex	2.23	0.29–17.39	0.443			
Clinical:						
1. Arterial hypertension	1.33	0.08–21.13	0.841			
2. Diabetes mellitus	1.36	0.21–1.59	0.124			
3. Hypercholesterolemia	1.34	0.10–18.13	0.828			
4. Active smoking	1.18	0.74–8.57	0.89			
Hair–scalp trace elements:						
1. Zinc (Zn)	1.03	1.01–10.5	0.006 *	1.03	1.01–1.05	0.001 *
2. Copper (Cu)	1.02	0.98–1.07	0.289			

Abbreviations: CI—confidence interval, OR—odds ratio. * statistically significant.

## Data Availability

The corresponding authors agree to share all data within 3 years following publication.
